# Lower-Extremity Muscle Strength Symmetry Assessment Through Isokinetic Dynamometry

**DOI:** 10.3390/life15020318

**Published:** 2025-02-19

**Authors:** Yuanyuan Ren, Sheng Zhou, Guangzhen Cheng, Yueqin Tang, Guangge Wang, Aming Lu

**Affiliations:** 1 Department of Basic Course, Suzhou City University, Suzhou 215104, China; yuanyuanren2021@126.com (Y.R.);; 2 Physical Education and Sport Science, Soochow University, Suzhou 215006, China

**Keywords:** lower extremity, muscle strength, symmetry, isokinetic

## Abstract

Objective: This study aimed to establish a dominant and non-dominant limb muscle strength evaluation model to evaluate the asymmetry of lower extremity muscle strength. Methods: The hip, knee, ankle flexors and extensors of 86 runners were evaluated separately in different contraction modes and at different movement speeds. A principal component analysis was used to establish a model for evaluating dominant and non-dominant lower extremity muscle strength and to comprehensively evaluate the asymmetry of lower extremity muscle strength. Results: Six main factors were present in both dominant and non-dominant indicators of lower extremity muscle strength, with dominant indicators of lower extremity muscle strength explaining 80.413% of the total variance and non-dominant indicators explaining 78.607% of the total variance. Conclusions: In a population of healthy male runners, there were differences in the symmetry of lower limbs in the comprehensive assessment model. The main contribution of the non-dominant side was the knee muscles, and the dominant side was the hip and knee muscles, so this difference should be considered in constructing future muscle strength evaluation models. It is critical to understanding the design and function of the human muscle system, and can reduce the number of meaningful tests we perform on diverse populations and help us reduce asymmetry.

## 1. Introduction

Muscle strength asymmetries have been a focus of research in recent years, and previous studies have investigated interlimb strength asymmetries in a range of populations. A large number of studies have confirmed the existence of asymmetry between limbs, and interlimb asymmetry places both lower extremities at high risk for sports injuries [1,2]. For example, a study used the muscle strength of a single joint to study the symmetry of lower-extremity muscle strength and found that the asymmetry of the quadriceps muscle and hamstring muscle was moderately positively correlated with the relevant indicators of running [3], and the strength asymmetry between limbs would harm the running performance [4]. However, the existing evidence is inconsistent. Other studies have shown that although there is an obvious asymmetry in the peak power of muscles during squat jumps (the asymmetry ratio reaches 9.7%), the asymmetrical muscle strength of the dominant and non-dominant limbs does not affect the performance of L-shaped running [5]. This contradictory result may be due to the different testing methods of individual joint muscles selected in different studies. Recent studies have questioned the ability of muscle strength to predict injury based on the peak torque (PT) of a single joint [6,7], and the results of one or a few muscle groups do not predict well the strength of other muscle groups [8,9]. However, there are complex relationships among various single-joint muscle strength evaluation indicators, which are not suitable for a simple superimposed treatment; therefore, a comprehensive multi-indicator evaluation method to define lower extremity asymmetry may be needed to develop a more robust calculation method and provide a methodological reference for future studies of lower extremity muscle strength.

Different from the single-indicator evaluation method, the multi-indicator comprehensive evaluation method could synthesize or integrate multiple indicator information to obtain a comprehensive evaluation value. The principal component analysis method is often used in literature to determine indicator weight and construct a multi-indicator comprehensive evaluation model currently [10,11,12]. The advantage of principal component analysis over other methods is that the determination of indicator weights is more objective, and it has many applications in the construction of comprehensive evaluation indicators in the field of sports [8,9,10]. For example, one study used principal component analysis as well as multiple regression to predict the climbing ability of climbers by anthropometric data (such as height, weight, body fat, muscle percentage, etc.), general upper limb strength and specific strength. The results showed that the three components of anthropometric data, upper limb general strength, and specific strength together explained 64.22% of the variance in climbing ability and that climbing ability was more closely related to upper limb specific strength [13]. Other indicators such as knee torque, quadriceps muscle, and hamstring muscle activation level were observed during walking, and waveform patterns in different gait cycles were extracted by principal component analysis to explain greater moment difference and longer muscle activity with higher principal components [14]. However, it is unclear which mode of contraction (centripetal or centrifugal) and angular speed of movement (fast or slow) of isokinetic muscle strength test results are more suitable for differentiating individuals with different strength levels, and whether it is possible to summarize the strength test results of different muscle groups of the lower extremity with a minimum number of muscle strength test indicators.

Therefore, the purpose of this study was to perform correlation analysis and principal component analysis on each indicator of lower extremity muscle strength, screen out representative indicators of lower extremity muscle strength, and then establish a dominant-side and non-dominant-side lower extremity muscle strength assessment model, respectively, providing a theoretical reference for future research on lower extremity muscle strength symmetry. Assuming that there is a strong correlation between the lower extremity muscle strength indicators of each joint of the lower extremity, and these indicators could be calculated by principal component analysis to obtain the dominant side and non-dominant side limb muscle strength evaluation models, and then the symmetry of the lower extremity muscle strength could be evaluated.

## 2. Methods

### 2.1. Subjects

Eighty-six healthy male non-professional runners aged 19–26 years with running habits (age 21.64 ± 2.58 years, height 179.47 ± 6.15 cm, weight 73.90 ± 10.39 kg) were recruited. Inclusion criteria: (1) running twice a week, more than 30 min each time, with a running distance of more than 15 km per week; (2) no lower-extremity injury and no regular strength training within six months. The study was approved by the Ethics Committee of Soochow University (No. SUDA20211227H03, 27 December 2021). The sample size was estimated using G-Power software (Version 3.1), with type I error α set at 0.05, effect size set at 0.3 for moderate effect, and statistical test power (Power) at 0.8, and the minimum sample size of 82 was calculated, taking into account the possible existence of a certain sample loss rate, and 86 subjects were finally included.

### 2.2. Experimental Protocol

- Muscle strength test

**Figure 1:**
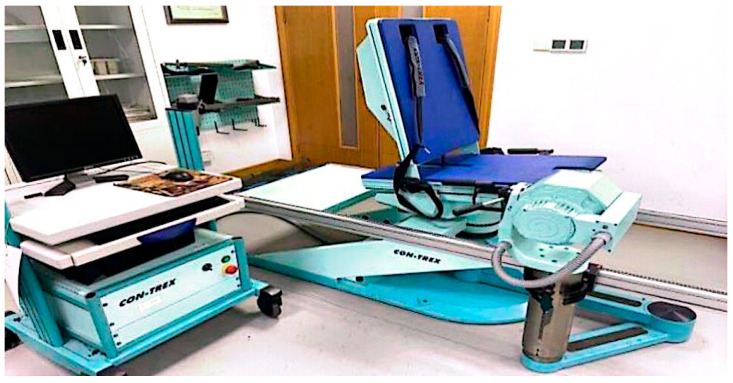
Isokinetic muscle strength test system.

The isometric muscle strength test system was used to test the flexor and extensor muscles of the subjects’ lower extremity hips, knees and ankles (Figure 1). The strength test was repeated at different angular speeds (fast and slow) and in different contraction modes (Concentric, Eccentric) [15]. In order to avoid the fatigue effect, each subject repeated the movements only 3–5 times after sufficient warm-up, and the rest time between each set was at least 120 s. During each test, the order of testing was also randomized for different angular velocities of movement (fast, slow), different contraction patterns (centripetal, centrifugal), and muscle groups (extensors and flexors). Each subject performed all training at the same time of day (±1 h) and in the same experimental environment (22 °C, 58% humidity). The peak torque values observed in different tests were recorded by researchers.

- Morphological test

Morphological measurements were performed on all subjects using auxiliary equipment such as tape measures, calipers, and weight scales, and subjects with obvious abnormalities in lower extremity morphology such as long and short legs were excluded.

### 2.3. Experimental Process

① Subjects signed an informed consent form and filled in basic personal information (height, weight, age, training years, injury history, etc.), and changed into tight-fitting shorts and sports shoes uniformly equipped in the laboratory. ② The subjects were tested for height, weight and other morphological information, and all tests were supervised by the same professional. ③ Subjects warmed up for 5–10 min at a speed of 6 km/h on the running platform. ④ After the warm-up, a professional explained and demonstrated the experimental test movements of the subjects, and the subjects practiced the test movements as required to get familiar with the test methods. ⑤ Subjects were randomly tested using the lottery method for muscle strength of the dominant and non-dominant lower extremities, and encouraging instructions were given by the tester during the test to increase the subject’s arousal to obtain maximum muscle strength. ⑥ After sufficient rest, the subjects performed the next joint of muscle strength testing.

### 2.4. Data Acquisition and Processing

The data of the muscle strength test were derived from the software that comes with the isokinetic plyometric test system, and the subsequent operation of the data was performed using Matlab software (Version R2021a) [15].

The assessment of lower-extremity muscle strength includes muscle contraction tests at different contraction forms and different angular velocities of movement. In many studies on the isokinetic muscle strength test of the lower extremity, the angular speed of 60 °/s is the slow angular speed most commonly used to test the maximum peak torque of hip, knee and ankle joints of the lower extremity [16,17,18]. One study tested the dorsiflexion and plantar flexors peak torque of the ankle joint at a speed of 60°/s [19], and the results show that there is greater reliability of peak torque values for this speed. The angular velocity of 60°/s is also a commonly used method to test the isokinetic muscle strength of the knee joint and hip joint [15,17]. In articles studying knee muscle strength, a fast angular velocity of 300°/s was chosen to measure knee flexor–extensor contraction moments because it is close to the velocity of a running motion [15,20,21], and this test method has been widely used in numerous publications [20,21,22,23,24]. A study reviewing the reliability of isometric measurements of hip flexion and extension strength found good reliability for hip flexor strength tests and hip extension strength tests at an angular velocity of 180°/s [25], which has also been widely used in previous studies [17,18,26,27]. The peak moment torque measured in plantarflexion and dorsiflexion of the ankle joint at 120°/s test speed has also been widely used [27,28,29]. In summary, the study summarized the indicators of flexor and extensor muscle strength of each joint of the lower extremity at different angular velocities (slow and fast) (Table 1).

### 2.5. Statistical Analysis

Kolmogorov–Smirnov was used to test the normality of data distribution. Pearson correlation analysis was conducted for each indicator of dominant and non-dominant muscle strength in 86 statistical samples to determine the correlation between different muscle indicators. The r values were interpreted qualitatively according to the definition of Hopkins et al. [30], with r = 0.00–0.09 being a slight correlation; r = 0.10–0.29 being a mild correlation; r = 0.30–0.49 being a moderate correlation; r = 0.50–0.69 is highly correlated; r = 0.70–0.89 is very highly correlated; and r = 0.90–0.99 is near perfect.

The 24 indicators of lower-extremity muscle strength were analyzed by principal component analysis (PCA), and the main indicators of the dominant and non-dominant side muscle strength evaluation model were constructed according to the eigenvalues and variance contribution rates. Then, principal component analysis was used to establish the comprehensive score of the dominant and non-dominant side muscle strength evaluation model, and the principal component with an eigenvalue greater than 1 was retained [8]. The symmetry indicator was calculated by the model score of the dominant and non-dominant muscle strength evaluation models (Z score) to obtain the comprehensive score of lower extremities muscle asymmetry. The symmetry index (SI) is used to evaluate the symmetry of muscle strength of the lower extremities, which is also the most commonly used index in symmetry evaluation studies. The calculation formula is as follows [31,32,33]: $SI=\left[2(Z_{1}-Z_{2})/(Z_{1}+Z_{2})\right]$, Z_1_ represents the non-dominant side, and Z_2_ represents the dominant side. The smaller the SI value, the better the symmetry of muscle strength on both sides, and the larger the SI value, the worse the symmetry between the lower extremity. Measurements were expressed as “mean ± standard”, *p* < 0.05 means significant difference, and *p* < 0.01 means extremely significant difference.

## 3. Results

**Figure 2:**
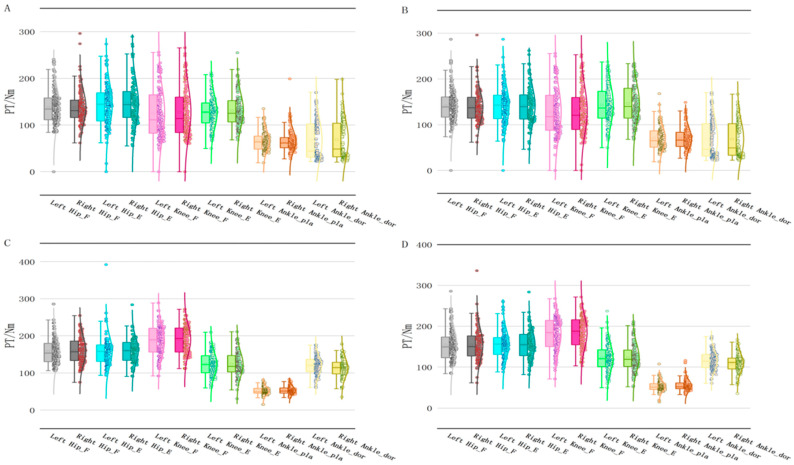
Peak torque of muscles in joints of lower extremities. Note: (**A**) represents peak torque of the lower extremity muscles in concentric-fast contraction mode; (**B**) represents peak torque of the lower extremity muscles in concentric-slow contraction mode; (**C**) represents peak torque of the lower extremity muscles in eccentric-fast contraction mode; (**D**) represents peak torque of the lower extremity muscles in eccentric-slow contraction mode. PT = Peak torque; Left/Right Hip_E/F = Left/Right Hip extensor/flexor peak torque; Left/Right Knee_E/F = Left/Right Knee extensor/flexor peak torque; Left/Right Ankle_pla/dor = Left/Right Ankle plantar malleolus/dorsal flexor peak torque.

A Kolmogorov–Smirnov test confirmed the normal distribution of the data (*p* > 0.05). From the descriptive results, it appears that there may be an imbalance in lower limb muscle strength between the dominant and non-dominant sides of the subject. As shown in Figure 2A, the peak torque of hip flexors, hip extensors, knee flexors, knee extensors and ankle extensors of the dominant lower extremity was higher than non-dominant lower extremity in Con_F. The peak torque of knee flexors, knee extensors and ankle extensors of the dominant lower extremity was higher than the non-dominant lower extremity in Con_S (Figure 2B). The peak torque of the hip extensor, knee flexor and ankle flexor of the dominant lower extremity was higher than non-dominant lower extremity in Ecc_F, while the peak torque of the hip flexor, knee extensor and ankle extensor of dominant lower extremity was lower than non-dominant lower extremity (In Figure 2C). The peak torque of knee flexors, knee extensors and ankle flexors of the dominant lower extremity in Ecc_S was higher than non-dominant lower extremity, while the peak torque of hip flexors, hip extensors and ankle extensors of the dominant lower extremity was lower than in the non-dominant lower extremity (Figure 2D).

**Figure 3:**
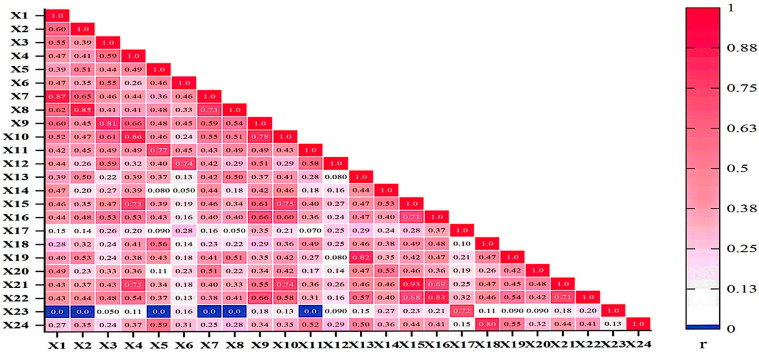
Correlation of non-dominant lower extremity muscle peak torque. Note: r denotes the correlation coefficient, which reflects the correlation between the peak torque indicators of non-dominant lower extremity muscle, with darker colors representing higher correlations. X_1–24_ = Con_F_Hip_F; Con_F_Hip_E; Con_F_Knee_F; Con_F_Knee_E; Con_F_Ankle_pla; Con_F_Ankle_dor; Con_S_Hip_F; Con_S_Hip_E; Con_S_Knee_F; Con_S_Knee_E; Con_S_Ankle_pla; Con_S_Ankle_dor; Ecc_F_Hip_F; Ecc_F_Hip_E; Ecc_F_Knee_F; Ecc_F_Knee_E; Ecc_F_Ankle_pla; Ecc_F_Ankle_dor; Ecc_S_Hip_F; Ecc_S_Hip_E; Ecc_S_Knee_F; Ecc_S_Knee_E; Ecc_S_Ankle_pla; Ecc_S_Ankle_dor.

There was a high correlation between each joint indicator of the non-dominant lower extremity in the Con_F and with the other indicators in the Con_S, Ecc_F, and Ecc_S contraction modes (*p* < 0.05) (Figure 3). However, ankle plantar flexor muscle strength in Ecc_S was not significantly correlated with hip flexor–extensor muscle strength and ankle plantar flexor muscle strength in Con_F and hip flexor–extensor muscle strength and ankle plantar flexor muscle strength in Con_S (*p* > 0.05).

**Figure 4:**
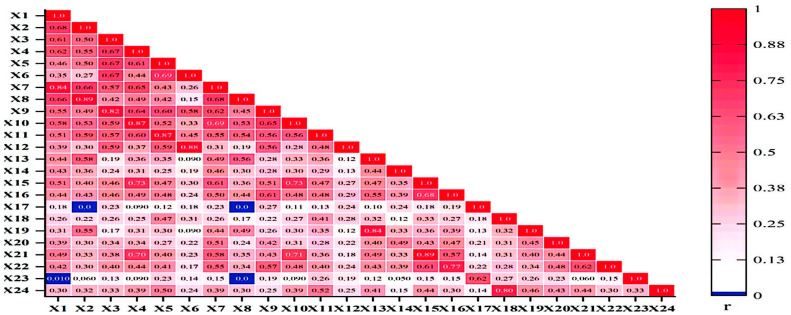
Correlation of dominant lower extremity muscle peak torque. Note: r denotes the correlation coefficient, which reflects the correlation between the peak torque indicators of non-dominant lower extremity muscle, with darker colors representing higher correlations. X_1–24_ = Con_F_Hip_F; Con_F_Hip_E; Con_F_Knee_F; Con_F_Knee_E; Con_F_Ankle_pla; Con_F_Ankle_dor; Con_S_Hip_F; Con_S_Hip_E; Con_S_Knee_F; Con_S_Knee_E; Con_S_Ankle_pla; Con_S_Ankle_dor; Ecc_F_Hip_F; Ecc_F_Hip_E; Ecc_F_Knee_F; Ecc_F_Knee_E; Ecc_F_Ankle_pla; Ecc_F_Ankle_dor; Ecc_S_Hip_F; Ecc_S_Hip_E; Ecc_S_Knee_F; Ecc_S_Knee_E; Ecc_S_Ankle_pla; Ecc_S_Ankle_dor.

There was a high correlation (*p* < 0.05) between each joint indicator of the dominant lower extremity in the Con_F (*p* < 0.05), as well as with other indicators in the Con_S, Ecc_F, Ecc_S contraction modes (*p* < 0.05) (Figure 4). However, there was no significant correlation between hip flexor strength in the Con_F and ankle plantar flexor strength in the Ecc_S (*p* > 0.05); hip extensor strength in the centripetal–fast contraction mode was not significantly correlated with ankle plantar flexor strength in the centrifugal-fast contraction mode (*p* > 0.05); There was no significant correlation between hip extensor strength in the Con_F and ankle plantar flexor strength in the Ecc_F (*p* > 0.05); There was no significant correlation between hip extension muscle strength in Con_S and ankle plantar flexor muscle strength in Ecc_F or Ecc_S (*p* > 0.05).

### Principal Component Analysis of Lower Extremity Muscle Strength Indicators

In order to test whether the muscle strength indicators of the lower extremity joints on the dominant side and the non-dominant side of the subject were suitable for principal component analysis, the Kaiser–Meyer–Olkin (KMO) test and Bartlett sphericity test were, respectively, performed on the original data. The KMO test is used to compare the simple correlation between variables, and Bartlett’s spherical test is used to test whether the correlation matrix is the identity matrix. When the KMO value is greater than 0.5 and the significance of the Chi-square statistics of the Bartlett sphericity test is less than 0.01, the data are suitable for principal component analysis.

As can be seen from Table 2, the KMO value of the non-dominant lower extremity muscle strength was 0.833 and the KMO value of the dominant side lower extremity muscle strength was 0.815. The approximate chi-square value of the Bartlett sphericity test was 1809.165 and 1839.031, respectively, and the significance level was *p* < 0.01. Principal component analysis could be performed on the data.

Principal component analysis was performed on all muscle strength indicators of the non-dominant side and the dominant side lower extremity, respectively, and the principle of eigenvalues greater than 1 was used to determine the principal components. As can be seen from Table 3, there were six factors with eigenvalues greater than 1 in the muscle strength indicators of the non-dominant lower extremity, with a cumulative contribution of 80.413%, and the main factors were composed of different testing methods acting on the knee muscles. There were six factors with eigenvalues greater than 1 in the dominant lower-extremity muscle strength indicator, with a cumulative contribution of 78.607%, while the main factors were composed of different testing methods acting on the knee and hip muscles. This indicates that the first six principal components represent 80.413% and 78.607% of the information of the original 24 indicators of non-dominant and dominant lower extremity muscle strength, respectively.

According to the score coefficient matrix of the principal component in Table 4, the scores of each principal component can be calculated, and the six principal components (Y1, Y2, Y3, Y4, Y5, Y6) and their contribution rates can be used to construct the comprehensive score Z_1_ and Z_2_ of the lower extremity muscle strength of the non-dominant (Z_1_) and dominant (Z_2_) sides, respectively.

The principal component scores of the non-dominant and the dominant lower-extremity muscle were calculated, respectively, according to the above calculation formula, and the non-standardized integrated score models of the lower-extremity muscle strength were calculated based on the contribution of each component, Z_1_ = 43.938 × Y_1_ + 9.953 × Y_2_ + 8.291 × Y_3_ + 7.155 × Y_4_ + 6.327 × Y_5_ + 4.749 × Y_6_, Z_2_ = 43.316 × Y_1_ + 10.099 × Y_2_ + 8.284 × Y_3_ + 6.734 × Y_4_ + 5.698 × Y_5_ + 4.475 × Y_6._

Ultimately, a comprehensive indicator for assessing lower extremity muscle strength symmetry was derived by calculating the symmetry index (SI), formulated as SI = 2 × (Z_1_ − Z_2_)/(Z_1_ + Z_2_), thereby enabling the evaluation of muscle symmetry in the lower extremities.

## 4. Discussion

The obtained results support the research hypothesis; this study found that there were some differences between the dominant and non-dominant lower extremity muscle strength, suggesting that there may be an asymmetry between the dominant and non-dominant lower extremity muscle strength in the subjects. This is consistent with the results of a previous study in which 24 healthy subjects were tested for isometric muscle strength and vertical jumping. It was found that there was asymmetry in the subjects’ knee extension muscle strength, and the subjects in the knee extension muscle strength asymmetry group had a higher ground reaction force asymmetry rate during vertical jumping [34]. This suggests that the presence of asymmetry in lower-extremity muscle strength may affect motor tasks, possibly due to differences in the level of leg muscle activation on the dominant and non-dominant side [34].

Further research results show that there is a significant correlation between the indicators, there is a significant correlation between the muscle strength indicators under the same contraction mode, and there is also a significant correlation between the muscle strength indicators under different contraction modes. The positive and negative of each correlation coefficient can reflect the correlation between each indicator: the negative number represents the negative correlation, and the positive number represents the positive correlation. This further shows that there is a complex correlation between these indicators, which is suitable for principal component analysis. The results of the principal component analysis showed that it was feasible to filter the major lower extremity muscle strength indicators to represent lower extremity muscle strength among the 24 original indicators. This method has been consistently accepted in previous studies on muscle strength [10,35,36]. Although previous studies have confirmed that a limited set of indicators can reflect overall muscle strength, they did not comprehensively assess muscle strength by considering antagonistic muscle groups. Our current study addresses this limitation. In this study, the lower-extremity muscle strength model not only incorporates various contraction forms of both agonist and antagonist muscles but also resolves the issue of reasonable weighting for multiple muscle strength indicators, thereby providing a more comprehensive evaluation of lower extremity muscle strength. In this study, six principal components were extracted from both the dominant and non-dominant lower extremity muscle strength models, and the cumulative contribution rates reached 80.413% and 78.607%, respectively, indicating that the basic characteristics of the dominant and non-dominant side lower extremity muscle strength levels could be reflected by these six principal components. In the principal component score model, the indicators of the six principal components of the two limbs were different. In the non-dominant lower extremity muscle strength model, the main indicators of Y1 included knee flexor in Con_S contraction mode, knee extensor in Con_S contraction mode, and knee flexor in Ecc_F contraction mode; the main indicators of Y2 included ankle extensor in Con_F and Con_S contraction mode; the main indicators of Y3 include the ankle flexor in Ecc_F contraction mode; the main indicators of Y4 include the hip flexor in Con_S contraction mode; the main indicators of Y5 include the ankle extensor in Con_F contraction mode and the ankle extensor in Con_S contraction mode; and the main indicators of Y6 include the hip extensor in Ecc_F contraction mode. In the dominant lower-extremity muscle strength model, the main indicators of Y1 include knee extensor in Con_F contraction mode and hip flexor in Con_S contraction mode; the main indicators of Y2 include ankle extensor in Con_F contraction mode and ankle extensor in Con_S contraction mode; the main indicators of Y3 include ankle flexor in Ecc_S contraction mode; the main indicators of Y4 include knee extensor in Ecc_S contraction mode; the main indicators of Y5 include the knee flexors in Ecc_S contraction mode; and the main indicators of Y6 include the ankle flexors in Ecc_S contraction mode. From the principal component analysis of the lower-extremity composite model, the six main components account for approximately 80% of the total variance. This suggests that these six components play a crucial role in the comprehensive evaluation of lower limb muscle strength and can provide substantial insight into the symmetry of muscle strength between both limbs. Therefore, it is hypothesized that under limited experimental conditions, these major components can be utilized to predict overall lower limb muscle strength as well as bilateral symmetry.

Similarly, previous studies also used principal component analysis to analyze various indicators, and showed that the overall limb and trunk muscle strength could be reflected by grip strength and knee extension force, which may be related to the few muscle strength indicators included in the study [35]. Another study has been conducted to comprehensively assess whole-body muscle strength by testing peak torque (the extensor and flexor muscles of the knee, hip, shoulder, and elbow joints in individuals) in a single joint by isometric testing methods, and the conclusions reached were similar to those of this study. Principal component analysis reveals that three factors explain 62.5% of the total variance, and the main factors were loaded by the different testing methods and strength variables for the muscles acting on the knee (first component), hip (second component) and arm joints (third component) [10]. Although PCA-based analyses have been conducted in previous lower extremity strength studies, variations in testing methods for individual joint muscles across different studies may have contributed to inconsistent results [8,9]. In contrast, our study comprehensively evaluated peak torque indicators of hip, knee, and ankle joint muscles under various contraction modes, including concentric/eccentric and fast/slow contractions, to minimize the influence of differing test methodologies. By performing principal component analysis on 24 bilateral lower-extremity muscle strength indicators, we selected representative primary indicators and established evaluation models for both the dominant and non-dominant sides. This novel approach has significant implications for understanding the design and function of the human muscular system. Moreover, previous research typically focused on the activation levels of specific or single lower extremity muscles and their relationships with certain factors, neglecting comparisons between dominant and non-dominant extremities. Notably, this study separately examined all muscles in the dominant and non-dominant lower extremities, assessing and comparing muscle symmetry. It assessed muscle symmetry in a healthy male runner population based on principal component scores and compared and revealed differences in bilateral lower extremity composite assessment models. This finding holds the potential to significantly reduce the number of tests required for athletes and diverse populations. Additionally, understanding the design and function of both dominant and non-dominant muscle systems across various populations could contribute to minimizing asymmetries and preventing injuries, among other advantages. However, this study has some limitations. First, although the sample size meets the basic requirements and the evaluation model could be constructed initially, it is necessary to expand the sample size and the sample range in order to further improve the evaluation effect of the symmetry model of lower limb muscle strength in the follow-up work. Secondly, the present study only tested the strength of lower-limb muscles under isometric contraction mode, and future research should also focus on the strength differences of muscles under more contraction conditions (e.g., isometric contraction), which will help to understand the relationship between the strength of individual joints and the symmetry of the whole lower limb muscle strength under different testing conditions. Future studies must integrate more comprehensive methodological approaches, including accounting for daily variability in individuals and enhancing the reliability of assessments, to ensure more robust conclusions.

## 5. Conclusions

This study assessed lower extremity muscle strength and symmetry in healthy male runners based on principal component scores of dominant and non-dominant lower-extremity muscle strength, revealing discrepancies in the composite evaluation model of the bilateral lower extremity. This novel finding enhances our comprehensive understanding of the structure and function of both dominant and non-dominant muscular systems across diverse demographics, thereby potentially mitigating the incidence of asymmetry. Furthermore, it has the potential to reduce the number of routine tests required for athletes and various populations.

## Figures and Tables

**Table 1:** Quantification of lower extremity muscle strength indicators.

Contraction Mode	Muscle Position	Definition
Concentric-Fast, Con_F	Hip_F	Hip flexor concentric contraction muscle peak torque at 180°/s angular velocity, denoted X_1_.
	Hip_E	Hip extensor concentric contraction muscle peak torque at 180°/s angular velocity, denoted X_2_.
	Knee_F	Knee flexor concentric contraction muscle peak torque at 300°/s angular velocity, denoted X_3_.
	Knee_E	Knee extensor concentric contraction muscle peak torque at 300°/s angular velocity, denoted X_4_.
	Ankle_pla	Ankle plantar malleolus concentric contraction muscle peak torque at 120°/s angular velocity, denoted X_5_.
	Ankle_dor	Ankle dorsal flexor concentric contraction muscle peak torque at 120°/s angular velocity, denoted X_6_.
Concentric-Slow, Con_S	Hip_F	Hip flexor concentric contraction muscle peak torque at 60°/s angular velocity, denoted X_7_.
	Hip_E	Hip extensor concentric contraction muscle peak torque at 60°/s angular velocity, denoted X_8_.
	Knee_F	Knee flexor concentric contraction muscle peak torque at 60°/s angular velocity, denoted X_9_.
	Knee_E	Knee extensor concentric contraction muscle peak torque at 60°/s angular velocity, denoted X_10_.
	Ankle_pla	Ankle plantar malleolus concentric contraction muscle peak torque at 60°/s angular velocity, denoted X_11_.
	Ankle_dor	Ankle dorsal flexor concentric contraction muscle peak torque at 60°/s angular velocity, denoted X_12_.
Eccentric-Fast, Ecc_F	Hip_F	Hip flexor eccentric contraction muscle peak torque at 120°/s angular velocity, denoted X_13_.
	Hip_E	Hip extensor eccentric contraction muscle peak torque at 120°/s angular velocity, denoted X_14_.
	Knee_F	Knee flexor eccentric contraction muscle peak torque at 120°/s angular velocity, denoted X_15_.
	Knee_E	Knee extensor eccentric contraction muscle peak torque at 120°/s angular velocity, denoted X_16_.
	Ankle_pla	Ankle plantar malleolus eccentric contraction muscle peak torque at 120°/s angular velocity, denoted X_17_.
	Ankle_dor	Ankle dorsal flexor eccentric contraction muscle peak torque at 120°/s angular velocity, denoted X_18_.
Eccentric-Slow,Ecc_S	Hip_F	Hip flexor eccentric contraction muscle peak torque at 60°/s angular velocity, denoted X_19_.
	Hip_E	Hip extensor eccentric contraction muscle peak torque at 60°/s angular velocity, denoted X_20_.
	Knee_F	Knee flexor eccentric contraction muscle peak torque at 60°/s angular velocity, denoted X_21_.
	Knee_E	Knee extensor eccentric contraction muscle peak torque at 60°/s angular velocity, denoted X_22_.
	Ankle_pla	Ankle plantar malleolus eccentric contraction muscle peak torque at 60°/s angular velocity, denoted X_23_.
	Ankle_dor	Ankle dorsal flexor eccentric contraction muscle peak torque at 60°/s angular velocity, denoted X_24_.

**Table 2:** KMO and Bartlett sphericity test.

		Non-Dominant	Dominant
KMO		0.833	0.815
Bartlett Sphericity	F	1809.165	1839.031
	df	276	276
	P	0.000	0.000

**Table 3:** Principal component characteristic value, variance contribution rate and cumulative variance contribution rate.

PC	Non-Dominant			Dominant		
	Eigenvalue	VCR (%)	CVR (%)	Eigenvalue	VCR (%)	CVR (%)
PC1	10.545	43.938	43.938	10.396	43.316	43.316
PC2	2.389	9.953	53.891	2.424	10.099	53.414
PC3	1.990	8.291	62.182	1.988	8.284	61.699
PC4	1.717	7.155	69.337	1.616	6.734	68.433
PC5	1.518	6.327	75.664	1.368	5.698	74.131
PC6	1.140	4.749	80.413	1.074	4.475	78.607

Note: PC = Principal components; VCR = Variance contribution rate; CVR = Cumulative variance contribution rate.

**Table 4:** Principal component score coefficient matrix.

	Non-Dominant						Dominant					
	PC1	PC2	PC3	PC4	PC5	PC6	PC1	PC2	PC3	PC4	PC5	PC6
X_1_	0.728	−0.264	0.033	−0.434	0.090	0.163	0.771	−0.085	−0.246	−0.043	0.234	0.118
X_2_	0.689	−0.209	−0.254	−0.222	0.253	−0.368	0.734	−0.220	−0.172	−0.418	0.294	0.091
X_3_	0.694	−0.305	0.365	0.037	−0.171	0.007	0.744	0.429	−0.215	0.035	0.105	0.012
X_4_	0.779	0.073	0.104	0.003	−0.379	−0.129	0.812	0.053	−0.302	0.046	−0.202	0.218
X_5_	0.646	−0.332	−0.33	0.413	−0.045	−0.106	0.754	0.403	0.059	−0.236	−0.138	−0.111
X_6_	0.517	−0.577	0.251	0.180	0.245	0.206	0.540	0.694	−0.033	−0.059	0.075	−0.287
X_7_	0.715	−0.281	0.004	−0.501	0.166	0.094	0.819	−0.194	−0.143	0.049	0.192	0.197
X_8_	0.672	−0.290	−0.229	−0.341	0.209	−0.380	0.687	−0.304	−0.242	−0.394	0.262	0.137
X_9_	0.814	−0.120	0.343	−0.007	−0.111	−0.097	0.770	0.300	−0.185	0.193	0.148	−0.034
X_10_	0.816	0.101	0.159	−0.121	−0.314	−0.144	0.779	−0.019	−0.323	0.064	−0.216	0.328
X_11_	0.642	−0.447	−0.197	0.305	−0.128	0.014	0.764	0.219	0.050	−0.272	−0.053	0.070
X_12_	0.504	−0.594	0.278	0.234	0.061	0.257	0.541	0.648	0.023	−0.043	0.153	−0.261
X_13_	0.653	0.354	−0.332	−0.071	0.354	0.007	0.622	−0.523	0.248	−0.173	0.108	−0.201
X_14_	0.541	0.371	0.106	−0.178	0.067	0.503	0.470	−0.261	0.071	0.197	0.381	−0.278
X_15_	0.806	0.293	0.123	0.025	−0.323	0.008	0.772	−0.158	−0.074	0.312	−0.363	0.063
X_16_	0.762	0.290	0.111	0.120	−0.086	−0.184	0.711	−0.175	0.036	0.307	−0.056	−0.268
X_17_	0.394	0.218	0.533	0.255	0.531	−0.131	0.257	0.228	0.494	0.454	0.368	0.367
X_18_	0.600	0.141	−0.458	0.419	−0.054	0.215	0.488	0.126	0.521	−0.318	−0.356	−0.060
X_19_	0.635	0.267	−0.418	−0.055	0.349	−0.051	0.556	−0.451	0.378	−0.245	0.137	−0.091
X_20_	0.564	0.218	0.015	−0.376	0.061	0.462	0.555	−0.192	0.301	0.241	0.097	−0.271
X_21_	0.781	0.322	0.080	0.005	−0.337	−0.017	0.717	−0.258	−0.095	0.309	−0.417	0.048
X_22_	0.752	0.379	0.053	0.048	−0.071	−0.165	0.661	−0.175	0.044	0.424	−0.109	−0.226
X_23_	0.227	0.350	0.505	0.365	0.482	−0.091	0.236	0.215	0.679	0.165	0.184	0.444
X_24_	0.630	0.052	−0.464	0.416	0.076	0.237	0.577	0.002	0.491	−0.338	−0.383	0.074

Note: PC = Principal components; X_1–24_ = Con_F_Hip_F; Con_F_Hip_E; Con_F_Knee_F; Con_F_Knee_E; Con_F_Ankle_pla; Con_F_Ankle_dor; Con_S_Hip_F; Con_S_Hip_E; Con_S_Knee_F; Con_S_Knee_E; Con_S_Ankle_pla; Con_S_Ankle_dor; Ecc_F_Hip_F; Ecc_F_Hip_E; Ecc_F_Knee_F; Ecc_F_Knee_E; Ecc_F_Ankle_pla; Ecc_F_Ankle_dor; Ecc_S_Hip_F; Ecc_S_Hip_E; Ecc_S_Knee_F; Ecc_S_Knee_E; Ecc_S_Ankle_pla; Ecc_S_Ankle_dor.

## Data Availability

The original contributions presented in this study are included in the article. Further inquiries can be directed to the corresponding author(s).
